# Impact of Delayed Time to Antibiotics in Medical and Surgical Necrotizing Enterocolitis

**DOI:** 10.3390/children10010160

**Published:** 2023-01-14

**Authors:** Katherine E. Chetta, Katherine G. Vincent, Bresney Fanning, Ashley B. Klumb, Justin A. Chetta, Allison M. Rohrer, Leslie H. Spence, Jeanne G. Hill

**Affiliations:** 1 Division of Neonatology, Department of Pediatrics, Medical University of South Carolina, 10 McClennan Banks Drive, Charleston, SC 29425, USA; 2 Department of Neuroradiology, Medical University of South Carolina, 96 Jonathan Lucas Street MSC 323, Charleston, SC 29425, USA; 3 Department of Pediatric Radiology, Medical University of South Carolina, 96 Jonathan Lucas Street MSC 323, Charleston, SC 29425, USA

**Keywords:** premature infant, necrotizing enterocolitis, antibiotics, pneumatosis

## Abstract

This study investigated whether delayed receipt of antibiotics in infants with necrotizing enterocolitis (NEC) is associated with disease severity. In this retrospective, single-center cohort study of infants diagnosed with NEC over 4 years, we compared the timing of antibiotic administration in infants (time order placed to time of receipt) in medical and surgical NEC. Cases were independently reviewed, then various clinical factors were compared. Of 46 suspected cases, 25 were confirmed by a panel of radiologists with good interrater reliability (ICC 0.657; *p* < 0.001). Delays in antibiotic receipt were 1.7× greater in surgical than medical NEC cases (*p* = 0.049). Every hour after order entry increased the adjusted odds of surgical NEC by 2.4 (1.08–5.23; *p* = 0.032). Delayed antibiotic receipt was more common in infants with surgical than medical NEC. Larger studies will be needed to investigate if optimizing antibiotic expediency could improve intestinal outcomes.

## 1. Introduction

Necrotizing enterocolitis (NEC) is a highly morbid intestinal disease that most commonly affects preterm infants. The causes of NEC are multifactorial, and risk factors include an altered gut microbiome, dysregulated intestinal immune signaling, formula feeding, poor intestinal perfusion, and pathogenic microorganisms [1,2]. Confirming an NEC diagnosis in clinical practice is complex [3]. A combination of clinical symptoms, laboratory values, and radiological findings are used to diagnose and prompt NEC treatment [4]. Clinical findings can be subtle, such as abdominal distension or lethargy, and at times obvious, such as bloody stools or bilious emesis. Additionally, radiographical findings such as pneumatosis, pneumoperitoneum, and portal venous gas are useful diagnostic features of NEC but are not universally present in all NEC cases [4]. Radiographic diagnosis is commonly uncertain in early or less aggressive NEC phenotypes. NEC can rapidly progress over time, and quickly lead to surgery and/or death for up to 50% of very low birth weight infants [5]. Indeed, there is potentially a wide range of clinical and individual factors that may be linked to disease severity.

NEC severity can be classified based on clinical presentation and radiological findings (such as modified Bell’s Criteria) or by end outcome (medical versus surgical NEC). It is unclear what underlying factors are associated with more severe cases. Large cohort studies using 16 s sequencing data from preterm infant stool indicate that NEC may be partially mediated by dysbiosis from certain sets of bacterial species [6,7], suggesting NEC disease has features that parallel sepsis. Pathogenic microbes may translocate into intestinal tissues that have poor integrity and leaky epithelial junctions, and this event triggers inflammation with similar features to early sepsis [2,8]. Pathogenic gut organisms are critical to developing NEC in animal models, without which, NEC cannot be induced [9]. Other commonalities between NEC and sepsis include empiric treatment with broad-spectrum antibiotics, common fulminant progression, similar laboratory markers of sepsis (elevated C-reactive protein and white blood cell counts, low platelets, etc.), and various associations with intestinal-linked pathogens [4,10]. It is presumed that if left untreated, mild or early cases of NEC rapidly develop end-organ intestinal damage—not unlike typical sepsis or other necrotizing pathologies in the adult population. Due to the key commonalities between sepsis and NEC, we aimed to investigate a factor in NEC that is known to mediate outcomes in sepsis: rapid treatment.

A major factor that modifies outcomes in pediatric sepsis is early, aggressive, and targeted therapy. Adult and pediatric sepsis guidelines recommend antibiotic administration to improve survival [11,12,13,14]. Because prompt treatment has been shown to improve sepsis outcomes, we hypothesized that rapid treatment of NEC is associated with mild NEC outcomes and potentially, delays are associated with requiring NEC surgery. We hypothesized that a longer time period, starting from a clinician diagnosis and ending at the start of administration of antibiotics, would be associated with surgical NEC as compared to medical NEC. To test this hypothesis, we examined a cohort of infants with confirmed NEC and compared the timing of antibiotic administration by medical and surgical outcomes.

## 2. Materials and Methods

### 2.1. Study Design and Population

This retrospective study reviewed the records of infants diagnosed with NEC from an internal database of diagnostic codes, 2018 through April 2022, at the Medical University of South Carolina (MUSC). Patients included in the study from 2018 to 2020 were diagnosed at MUSC Children’s Hospital, and those from 2020 to 2022 at the MUSC Shawn Jenkins Children’s Hospital, an 82-bed facility with a Level IV neonatal intensive care unit (NICU) that opened in the spring of 2020. The study was approved by the MUSC institutional review board (protocol number 00104755).

Because searches relying on diagnostic codes can be inaccurate [15], all possible cases of NEC identified in the electronic database were confirmed by a blinded panel of radiologists who reviewed the diagnostic radiographs. This was a critical step because non-NEC cases tend to introduce variation in pathology and treatment modalities, especially in single-center NEC studies [16]. Radiographically questionable cases were included if they met a certain set of clinical criteria. Radiologists’ ratings were compared using interrater reliability.

### 2.2. Independent Radiographic Review

Two pediatric radiologists and one double board-certified adult radiologist independently reviewed each patient’s initial diagnostic abdominal plain film and evaluated findings in three domains: pneumatosis, portal venous gas, and pneumoperitoneum. Radiologists used a 3-point scale to categorize image findings based on their degree of certainty for evidence of NEC: 0 = not present, 1 = maybe present or questionable, or 2 = definitely present. Each radiologist was provided a list of radiograph accession numbers, which included one abdominal radiograph per patient. Due to the limited number of studies, no ultrasounds were considered in the initial screening. The radiograph selected was performed on the day of NEC diagnosis, and if multiple radiographs were available that day, the image obtained immediately prior to obtained blood cultures was used. Radiologists reviewed images while blinded to the patient’s individual clinical course, original reads, and treatment course.

Grade 2 ratings were considered confirmed cases of NEC and included in the analysis, as were those with surgical findings consistent with NEC (regardless of grade). Questionable (grade 1) images were also included in the analysis if they met certain prespecified clinical criteria. These included the presence of bloody stool plus grade 1 pneumatosis or grade 1 pneumatosis associated with other radiographic findings in other categories. For example, if the radiologist indicated questionable (grade 1) in both portal venous gas and pneumatosis OR questionable pneumatosis and pneumoperitoneum, this was included as confirmed NEC. Infants with presentations consistent with spontaneous intestinal perforation (SIP) were excluded from the study.

### 2.3. Electronic Medical Record Review

Next, the electronic records were reviewed for demographic, peripartum, neonatal risk, and factors related to the time of NEC diagnosis. All factors were grouped by the presence or absence of surgical intervention for comparison. Demographic factors included maternal age, insurance status, presence of pre-eclampsia, diabetes, obesity, and COVID-19 status. Peripartum factors included type of delivery, fetal growth restriction or intrauterine growth restriction status, antenatal steroids, maternal antibiotics, chorioamnionitis, and prolonged preterm rupture of membranes. Neonatal risk factors included gestational age, birth weight, gender, race, multiple gestations, small for gestational age status, chromosomal anomalies, and major congenital anomalies. Factors at the time of diagnosis included radiographic findings, clinical presentation, timing of symptoms, liver or abdominal ultrasound, age at diagnosis, weight at diagnosis, use of formula, human milk or bovine hydrolyzed fortifier (HMF) (Similac ^®^, Abbott Nutrition) 4 days prior to diagnosis, intubation during NEC course, inotrope/pressor use, acute kidney injury (by KDIGO criteria [17]), patent ductus arteriosus (PDA) score, presence of a central line, C-reactive protein (CRP), platelet count, presence of cholestasis, culture and antibiotic order time, antibiotic administration, type of feeding, and culture results. Time to antibiotics was defined as the initial antibiotic order time minus the nurse-documented administration time at which the infant began receiving the infusion. The initial order time represents a practical surrogate datapoint for time of clinician diagnosis. Antibiotics used for all cases included vancomycin (10–20 mg/kg per I.V. dose q 8–24 h) and piperacillin-tazobactam (50–100 mg/kg per I.V. dose q 6–8 h), and dosing was dependent on weight and age. Outcome data included disposition (death, discharge to home or transfer), grade of bronchopulmonary dysplasia, and length of stay.

### 2.4. Statistics

Continuous variables were compared with independent *t*-tests between medical and surgical groups. Levene’s test for equality of variances was used, and then the appropriate 2-tailed *p*-values were selected. Normality was tested with Shapiro–Wilk tests. Nominal variables were compared with Chi-squared using Cochran’s and Mantel–Haenszel statistics when needed, and Fisher’s exact 2-sided significance. After initial comparison, T-test or Fisher’s exact variables significant to at least *p* = 0.2 were used for a final binary logistic regression model, which included gestational age, age at diagnosis, time to antibiotics, and medical vs. surgical NEC as a binary outcome. Correlation coefficients were determined using Fleiss interrater correlation with exact statistics. Statistics were performed using SPSS v. 27. Images were created in Prism (Graphpad ^®®^, Jolla, CA, USA). Significance was *p* < 0.05.

## 3. Results

**Figure 1:**
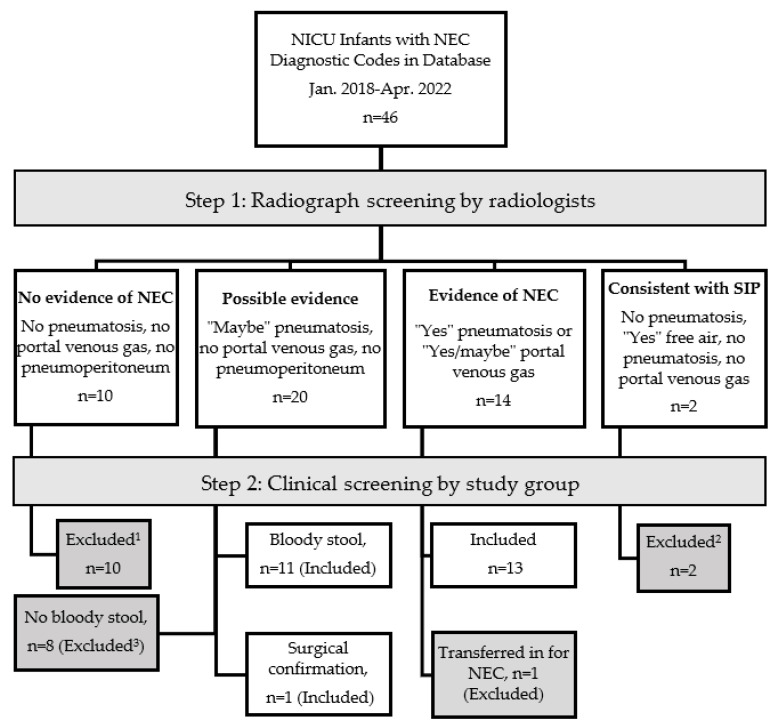
Flow diagram of necrotizing enterocolitis (NEC) selection from initial diagnoses code to end cohort included for analysis. EMR = electronic medical record, Dx = diagnosis, SIP = spontaneous intestinal perforation, ^1^ clinical course consistent with mild modified Bell Stage 1 or another non-NEC diagnosis, ^2^ SIP exclusion also considered relevant clinical factors (day of life, lack of feeds), ^3^ mild clinical courses without hemodynamic instability.

At a single institution, 46 infants were identified by diagnostic codes for NEC. After radiologist review and clinical/pathological criteria examined, NEC was confirmed in 25 of the 46 cases as demonstrated in the flow diagram (Figure 1).

NEC was confirmed by radiograph alone in 14 cases. Of the possible NEC radiographic cases (grade 1), and following clinical criteria, the presence of bloody stool confirmed 11 of the 20 possible cases. Infants who were ruled out by radiology (Grade 0) did not require surgery. All cases excluded were clinically reviewed. Nearly all excluded cases had symptoms consistent with modified Bell stage 1. One exception was included: a case of NEC with possible pneumatosis without bloody stool was later confirmed when surgery revealed a necrotic bowel. The demographics of infants with confirmed medical or surgical NECs are provided in Table 1 and the maternal and peripartum factors are seen in Table 2. Most infants were preterm except for two term infants who had congenital anomalies.

The 25 confirmed NEC cases were categorized as medical (16 [64%]) or surgical (9 [36%]) NEC for comparison. Maternal and peripartum features did not differ significantly between medical and surgical NEC cases. Infant characteristics, laboratory findings, other imaging studies, and other factors at the time of NEC diagnosis were obtained (Table 3).

At the time of diagnosis, 20% of all NEC cases received ancillary radiology studies such as abdominal and/or liver ultrasound. Compared with infants with medical NEC, infants with surgical NEC had significantly greater use of inotropes within 24 h of diagnosis and intubation (*p* < 0.001 and *p* = 0.005, respectively). The surgical NEC group was younger (16 v 29 days) and had lower platelet levels two days after diagnosis, but these findings did not reach significance (*p* = 0.1, *p* = 0.07). The number of positive blood cultures in each group was not different. Most infants (80%) received some human milk immediately before NEC diagnosis, and most infants in each group were on predominantly mother’s milk (at least 65% in each group).

**Figure 2:**
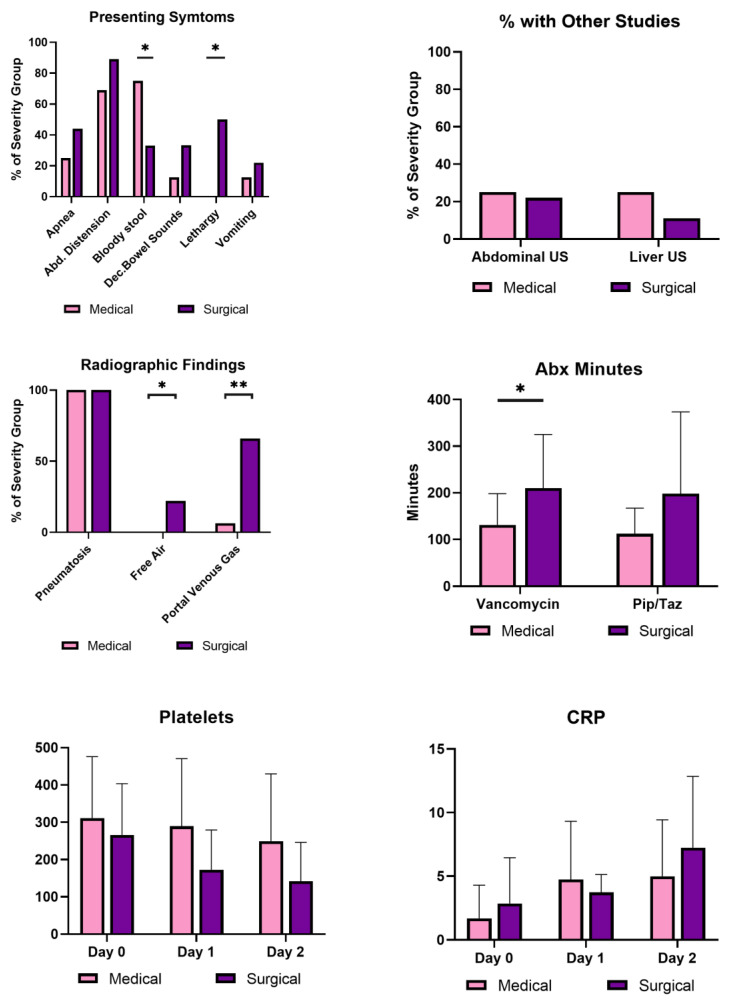
Comparison of clinical, laboratory, and antibiotic factors correlating to mild and severe NEC. Factors at diagnosis between medical (light) and surgical NEC (dark). C-reactive protein and platelet values: Day 0 is the day of NEC diagnosis. Fisher’s Exact test compared nominal variables, and *t*-tests compared continuous variables. Platelet units: 10^9^/L; CRP units: mg/dL. Significance was indicated by * *p*-value < 0.05, and ** *p*-value < 0.01.

The surgical group had significant differences in presenting symptoms, laboratory values, and time to receipt of antibiotics to the medical group (Figure 2). They presented more often with lethargy (55% v 0%) but less often with bloody stool (33% v 68%) than the medical group (*p* < 0.05). As to radiographic findings, they showed significantly more portal venous gas (*p* < 0.05) and pneumoperitoneum (*p* < 0.001) on initial radiography than the medical group.

It took longer for the surgical group to receive antibiotics (Table 4). The difference was significant for vancomycin (*p* = 0.032) but not for piperacillin/tazobactam (pip/taz). Definitive delays >2 h were documented more frequently in the surgical NEC group, but the difference was not significant (*p* = 0.21). Antibiotic times per patient are demonstrated in the Supplemental graphs. Outcomes are seen in Table 5 and there were five deaths (all in the surgical group), making up 20% of all confirmed NEC and 55% of the surgical NEC population.

In a binary logistic regression model of various clinical factors, including gestational age, and age at diagnosis, increased odds of surgical NEC correlated significantly with time (hours) to vancomycin with an adjusted estimate of 2.4 [1.08–5.23]; *p* = 0.032 but not time to pip/taz 1.63 [0.84–3.2]; *p* = 0.15. Two of the 25 cases had other antibiotics in addition to vancomycin and piperacillin. Every additional 30 min that passed after ordering any antibiotics increased the odds of having surgical disease 4.8 (1.09–23, *p* = 0.049) times.

To further verify the significance of antibiotic timing, the research team examined the eight cases of possible NEC that were excluded due to the absence of bloody stools. Four cases were subsequently included based on multiple clinical and radiographic findings by a consensus (medical n = 19, surgical n = 9). Expanding findings to include possible radiographic NEC without bloody stools maintained the significance of time to any antibiotic (*p* = 0.042), vancomycin (*p* = 0.033), and pip/taz (*p* = 0.12).

In a review of 46 cases, all radiologists agreed substantially with each other in rating their certainty about the presence or absence of radiographic findings (Fleiss multi-rater Kappa: 0.657; *p* < 0.001, Table 6). All Kappa values listed here are significant to *p* < 0.001. The highest kappa coefficients were seen with grade 0 (“not present”) and grade 2 (“definitely present”) and the lowest, indicating moderate agreement, with grade 1 (“possibly/ maybe present”). Among radiologists, grade 1 had lower agreement values than grade 0 or grade 2. For findings of pneumatosis, pneumoperitoneum, and portal venous gas, agreement was highest for pneumoperitoneum. Of all findings, agreement was lowest, but still moderate, for pneumatosis.

## 4. Discussion

Surgical NEC has a mortality rate of 20–50%, therefore identifying any factors contributing to NEC severity could lead to lives saved [18,19]. NEC treatment empirically targets gut pathobionts and has historically consisted of broad-spectrum antibiotics with Gram-positive, Gram-negative, and anaerobic coverage [20]. In general, there are few studies comparing antibiotic regimens in NEC [21,22,23,24]. While no study, to the best of our knowledge, has examined antibiotic delays as a risk factor for NEC severity. Early and rapid antibiotic treatment improves survival in adult and pediatric sepsis [14,25,26,27]. Here, we report that antibiotic administration delays were associated with surgical outcomes of NEC. Our findings suggest a modifiable factor, namely, rapid antibiotic administration, may mitigate NEC severity.

To accurately identify NEC for inclusion in the analysis, all diagnoses of NEC were examined and then externally reviewed to confirm the radiographic hallmarks of NEC. The radiologists reviewed all 46 cases and significantly agreed with each other across the three domains of findings. Our findings are similar to previous studies showing high interrater agreement among radiologists in NEC diagnosis [28]. The category that was tied to the highest agreement among radiologists was portal venous gas, and the lowest, but still moderate agreement, was pneumatosis. The radiologists also agreed, when pneumatosis was ‘maybe’ present. Maintaining moderate agreement even in uncertainty may indicate that there are limitations of the study itself to detect some cases of NEC, and less to do with the radiologist’s adopted style, ability, or expert level. Because pneumatosis is the most common pathognomonic finding in NEC, radiographic confirmation often prompts treatment. Uncertainty in these findings may lead to affecting the timing of the decision to treat. More sensitive and specific imaging or a new modality should be pursued to aid the diagnosis of NEC. A biomarker for NEC is needed to better predict the presence of early or subtle presentations of NEC, give insight into its severity, and promote early treatment [29]. If abdominal radiographs are often ambiguous for pneumatosis, over other findings, then better radiologic diagnostic tools are needed. Tools such as grayscale, Doppler, and contrast-enhanced ultrasound or other novel modalities may serve to enhance early diagnostic capabilities [30,31]. Once a diagnosis is made, treatment should proceed rapidly.

In this study, the time necessary to administer antibiotics in practice was longer than we would have anticipated, with the averages of both groups exceeding 2 h with large variances. Multiple patient or system/operational factors could contribute to delays. From patient-related factors, delays could occur from intravenous access difficulty in an already critically ill patient with poor perfusion. Interestingly, more central lines were in place for medical (19%) than surgical (0%) NEC cases. Although this finding is not statistically significant, having a central line in place at the time of diagnosis may have removed intravenous access issues and decreased time to antibiotic treatment, leading to a milder outcome. Systemic/organizational factors may be relevant in other units beyond our own. Burdens on staff including more complex patients, lower nurse-to-patient ratios, supply issues, and other barriers may be present affecting treatment expediency. Direct, closed-loop communication to bedside nursing at the time of diagnosis may also be key to expedient delivery of care.

Traditional procedural steps for diagnosis, including drawing cultures (blood, urine, and cerebral spinal fluid) before administering antibiotics, could be factors at play. Interestingly, most blood cultures in NEC often do not reveal a causative organism or guide treatment [19,32]. Questions remain regarding what cultures are necessary to guide the treatment of NEC [33]. Because diagnosing NEC is complex, we did not attempt to evaluate if an NEC diagnosis could have been made earlier than the decision to treat. We aimed to eliminate this diagnostic timing variability across events by only observing delays after the decision was made by the evidence of documented order times. Future studies could attempt to incorporate diagnostic delays in the model for rapid treatment by studying patient vital sign data with machine learning or including emerging early-detection tools such as abdominal electro-gastrography [34,35].

Antibiotic delays were not the only factor differing in medical and surgical NEC disease. To evaluate other differences between groups for modeling the binary outcomes, demographics and maternal and peripartum factors were highlighted. First, we did not see major differences in demographic data, maternal or peripartum (delivery) factors. This may be explained by our small sample size and possible effect sizes that are too small to detect in this study. Yet, unanticipated differences were seen in clinical factors at the time of diagnosis. C-reactive protein (CRP) levels stabilized the day after diagnosis in the medical NEC group but continued to rise in surgical NEC. No significant differences were found between individual days of CRP levels, but an average 3-point difference was noted 24 h after diagnosis (*p* = 0.10). The medical group had a lower average CRP than the surgical NEC 2 days after diagnosis, which may suggest an adequate response to antibiotic treatment, resolution of bacterial infection, and decreased inflammation [36]. The continuous rise in CRP 2 days after diagnosis in the surgical NEC group could be an early indicator of medical treatment failure. A study by Schmit et al. reported that CRP concentrations that increased at least 2.2 mg/dl in the first 48 h after an adult diagnosis of sepsis was associated with ineffective antibiotic therapy with a sensitivity of 77% and a specificity of 67% [37]. Whether CRP could be used as an indicator of treatment response in NEC could be further studied.

Platelet values fell in the surgical NEC group with the lowest platelets two days after diagnosis (*p* = 0.07). While not quite significant, this finding is consistent with previous studies suggesting that rapidly decreasing platelet counts herald poor outcomes [38,39]. According to Ververdis et al., platelet counts falling by >100 × 10^9^/L per day are strongly associated with the presence of necrotic bowel [39]. In this study, most (7/9) infants with surgical NEC required inotropes within 24 h of diagnosis but inotropes were rarely (1/16) used in the medical NEC group. Additionally, all infants with surgical NEC were intubated or required intubation within 24 h of diagnosis, while less than half of the infants in the medical group needed intubation. These findings are in accordance with the phenotypic nature of severe surgical disease. These factors also suggest that surgical NEC has apparent findings around the time of NEC diagnosis, and surgical NEC often requires rapid intervention. More information is needed to determine if the clinical features present around NEC diagnosis can predict severe phenotypes.

Most symptoms were not significantly different at the time of diagnosis with two exceptions; bloody stools were more frequent in the medical NEC group, and lethargy was more common in the surgical NEC group. This finding might be an artifact from pre-selected criteria that included bloody stool as the “tie-breaker” for all grade 1 radiographic NEC cases, and grade 1 questionable cases were mild in nature in this study. While we intentionally used this approach to catch as many NEC treated in the clinical environment, it likely inflated the mild cases with this finding. Alternatively, another reason may exist for finding bloody stools in the mild group. This readily visible sign might have prompted earlier treatment in the clinical course. Bloody stools are a more observable finding than lethargy for preterm infants, and if recognition was key, this may have led to milder outcomes by the mechanism of earlier treatment. On another note, blood in stools alone may also be reflective of cow’s milk-induced allergic colitis or other pathologies distinct from NEC [40,41]. Subtle signs, such as lethargy, were observed in surgical NEC groups, and this feature may have affected the time to recognition of NEC. While early recognition generally improves outcomes in treatable, progressive diseases as a general rule, more studies are needed to report the link between early detection and reduced NEC severity. Intriguingly, in both medical and surgical NEC, 65% of infants had received mother’s milk in the four days before the NEC diagnosis. While mother’s milk continues to be the singular best intervention to reduce NEC [42,43,44] more work is needed to determine why modern NEC continues to occur on human milk-based diets [45].

### Limitations of the Study

This single-center study has limitations. While the data shows that the time from diagnosis to antibiotic receipt was shorter in cases of medical NEC than surgical NEC, the study is limited by its modest sample size. A sizable population of non-NEC cases was excluded by radiographic review. More than half of all excluded cases generated by EMR diagnosis alone had only presented mild non-specific symptoms and were consistent with Bell Stage 1. There were four excluded cases within the radiographic category of ‘possible pneumatosis’ that did not have bloody stools, but that the research team consensus agreed reasoned to be early NEC based on clinical signs. When these four cases were included in the model, the significance of the timing of antibiotics was unchanged, adding to the veracity of this study.

Most importantly, due to the limited number of cases and subsequent loss of power, we were unable to account adequately for severity of illness as a confounder of NEC outcomes. There was a difference in the severity of NEC as noted by the frequency of intubation and inotrope use in the surgical group, but it is unclear if this is a confounding factor or in the causal pathway for fulminant disease. Worsening severity may be a potential downstream effect of delayed antibiotics, and we did not attempt to account for these factors. However, the signs of a lethargic, rapidly decompensating infant needing cardiorespiratory support may also prompt response teams to administer antibiotics more rapidly.

We note that at our center, vancomycin and pip/taz is the preferred treatment for NEC; however, we appreciate that the most commonly used NEC antibiotic regimen consists of ampicillin/gentamicin combination therapy with or without metronidazole [24]. This difference could limit the generalizability of this study. We found that the time to vancomycin but not pip/taz was significantly associated with the need for surgery. This is an interesting finding because when blood cultures are positive in NEC, an organism frequently isolated is coagulase-negative staphylococcus (CONS) [19]. The importance of the virulence of CONS in NEC pathophysiology is unknown. In general, the specific importance of vancomycin is questionable because receipt of vancomycin and pip/taz were highly collinear in the model. An interventional trial is needed to determine if vancomycin or another antibiotic combination is a superior choice for empiric coverage in NEC.

We hope this study prompts future datasets to report variables, such as antibiotic timing, within a multi-institutional framework. Therefore, the discussion points of this study are generally limited to this cohort.

## 5. Conclusions

In summary, this study identified that antibiotic timing and administration may be a factor in NEC outcomes. Because there is a high correlation between NEC surgery and death, focusing efforts on rapid antibiotic treatment could increase survival after NEC diagnosis. A larger multi-center study could validate the influence of timing seen in this study.

## Figures and Tables

**Table 1:** Demographics by severity of necrotizing enterocolitis (NEC).

Demographics ^1^	All NEC N = 25	Medical n = 16	Surgical n = 9	*p*-Value
Gestational age (wk)	28.5 ± 4.2	28.9 ± 4.3	29.6 ± 4.1	0.34
Birth Weight (g)	1184 ± 787	1132 ± 880	1277 ± 625	0.095
Sex, male	14 (56)	8	6	0.35
Multiples	5 (20)	2	3	0.54
Congenital anomalies	3 (12)	1	2	0.91
Maternal Age (y)	31 ± 7	31 ± 7	31 ± 8	0.92
Race Black White Other Not given	12 (48) 8 (32) 2 (8) 2 (8)	8 5 1 1	4 3 1 1	0.44
Ethnicity, Hispanic	1 (4)	1	0	
Insurance Medicaid Private Other	3 (12) 18 (72) 4 (16)	1 12 3	2 6 1	0.46

^1^ Categorical values are given as No. (%) and continuous values as (mean ± SD). GA = gestational age.

**Table 2:** Maternal and peripartum factors.

Factors at Diagnosis ^1^	All NEC N = 25	Medical n = 16	Surgical n = 9	*p*-Value
Delivery Type, (c/s)	16 (65)	11 (69)	5 (55)	0.51
Maternal diabetes	7 (28)	4 (25)	3 (33)	0.50
Maternal obesity	8 (32)	4 (25)	4 (44)	0.51
Pre-eclampsia	6 (24)	4 (25)	2 (22)	0.88
Covid19	1 (4)	0	1 (11)	0.17
Chorioamnionitis	1 (4)	1 (6)	0	0.44
Maternal abx	14 (56)	10 (62)	4 (44)	0.38
Antenatal Steroids Complete Partial None	12 (48) 6 (24) 7 (28)	5 3 1 (6)	7 3 6	0.38
PPROM	7 (28)	4 (25)	3 (33)	0.66
IUGR	6 (24)	4 (25)	2 (22)	0.88

^1^ Pearson, Chi-square tests (2-sided), significance *p* < 0.05. COVID-19 diagnosed by PCR within 2 weeks of delivery. Maternal antibiotics included all antibiotics given in labor (other than a single dose for c/s). Abx = antibiotics; c/s = caesarian section, PPROM = preterm prolonged rupture of membranes >18 h and <36 weeks gestational age.

**Table 3:** Factors at Diagnosis.

	All NEC ^1^ N = 25	Medical n = 16	Surgical n = 9	*p*-Value
Weight	1420 ± 889	1476 ± 1017	1321 ± 640	0.60
Age (d)	24 ± 21	29 ± 24	16 ± 12	0.17
Other Studies				
Abdominal US	6 (24)	4 (25)	2 (22)	0.8
Liver US	5 (20)	4 (25)	1 (11)	0.36
Lab Values				
CRP (day 0) mg/L	2.5 ± 3.1	2.3 ± 2.9	2.9 ± 3.6	0.69
CRP (day 1) mg/L	5.7 ± 4.0	6.4 ± 4.6	3.7 ± 1.4	0.10
CRP (day 2) mg/L	6.5 ± 4.8	6.1 ± 4.6	7.2 ± 5.6	0.69
Platelets (day 0) 10^9^/L	308 ± 145	331 ±148	266 ± 137	0.28
Platelets (day 1) 10^9^/L	261 ± 156	302 ±162	142 ± 104	0.06
Platelets (day 2) 10^9^/L	212 ± 165	250 ± 182	107 ± 40.3	0.07
Inotropes ^2^	8 (32)	1 (6)	7 (78)	<0.001
Intubation ^3^	16 (64)	7 (43)	9 (100)	0.005
AKI (by KDIGO)	3 (12)	1 (6)	2 (22)	0.23
Central access	3 (12)	3 (19)	0	0.23
PDA (n = 23 ECHOs)	3 (21)	2 (17)	1 (11)	0.83
Positive blood cultures	5 (20)	2 (13)	3 (33)	0.21
Diet Any formula Unfortified HM Fortified HM (HMF)	5 (20)	4 (25) 3 (15) 9 (45)	1(11) 0 8 (100)	0.62 0.24

^1^ Including all confirmed NEC findings based on blinded and clinical review. ^2^ Inotropes at time of diagnosis or in the following 24 h (included dopamine and epinephrine). ^3^ Intubated within 24 h before or after diagnosis. Central access at time of diagnosis. PDA determined by ECHO scoring criteria ≥ 7 or pediatric cardiology report. Human milk = HM, human milk fortifier = HMF (hydrolyzed, bovine), US = ultrasound, ECHOs = echocardiography reports, CRP = C-reactive protein, PDA = patent ductus arteriosus, KDIGO = Kidney Disease Improving Global Outcomes criteria. *p*-value significance < 0.05, comparing medical and surgical groups via *t*-tests or rank sum when applicable.

**Table 4:** Timing of antibiotic treatment by disease severity.

Antibiotics	All NEC N = 25	Medical n = 16	Surgical n = 9	*p*-Value
Time to any antibiotic ^1^ (min)	151 ± 96	122 ± 58	204 ± 129	0.049
Time to vancomycin (min)	160 ± 93	131 ± 67	210 ± 115	0.032
Time to piperacillin-tazobactam (min)	143 ± 93	112 ± 56	198 ± 176	0.15

^1^ All infants received both vancomycin and piperacillin/tazobactam (pip/taz) in this study. Adjusted for BW and age in binary linear regression model. Continuous variables are given as means ± SD. Radiographic findings were also compared.

**Table 5:** Outcomes: All NEC.

Outcomes	All NEC n = 25
Discharge Home	18 (72)
Death	5 (20)
Transferred to another facility	2 (8)
NEC Totalis ^1^	3 (13)
Any bronchopulmonary dysplasia	16 (64)
Severe bronchopulmonary dysplasia ^2^	10 (40)

^1^ As determined by surgical report. ^2^ Defined as requiring invasive positive pressure ventilation.

**Table 6:** Radiologist Intraclass Correlation (ICC) by Fleiss multi-rater Kappa.

	All Radiologists (3 Raters)	Interpretation of Agreement
All studies (N = 46)	0.657 (0.585–0.730)	Substantial
ICC by findings		
Pneumatosis	0.454 (0.336–0.573)	Moderate
Portal venous gas	0.629 (0.492–0.765)	Substantial
Pneumoperitoneum	0.719 (0.589–0.879)	Substantial
ICC by Certainty		
No NEC	0.739 (0.642–0.843)	Substantial
Maybe NEC	0.412 (0.316–0.508)	Moderate
Definitely NEC	0.763 (0.666–0.859)	Substantial

All Kappa coefficients are significant to *p* < 0.001. Fleiss ratings were interpreted on a scale of: <0 = Poor, 0–0.2 = slight, 0.21–0.40 = fair, 0.4–0.6 = moderate, 0.61–0.80 = substantial, 0.81–1.0 = almost perfect agreement. ICC = intraclass correlation coefficient.

## Data Availability

Not applicable.

## Supplementary Materials

The following are available online at https://www.mdpi.com/article/10.3390/children10010160/s1, Figure S1: Plot of Vancomycin Delay in Minutes by Medical or Surgical NEC Group; Figure S2: Plot of Piperacillin/tazobactam Delay in Minutes by Medical or Surgical NEC Group; Figure S3: Plot of Average Antibiotic Delay in Minutes per Infant by Medical or Surgical NEC Group.
