# Supplementary material for: Impact of Delayed Time to Antibiotics in Medical and Surgical Necrotizing Enterocolitis
Source: Children (Basel). 2023 Jan 14;10(1):160. doi: 10.3390/children10010160 (PMC9856867; doi:10.3390/children10010160)
Supplement: Supplementary file 1 [file children-10-00160-s001.zip › children-2116208-supplementary.pdf]

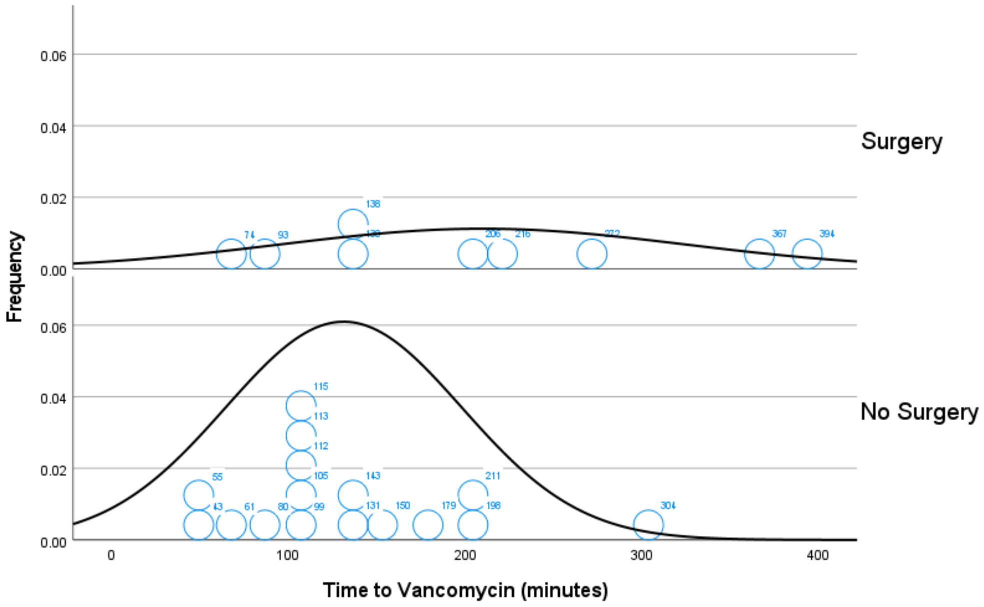

Figure S1. Histogram of minutes to vancomycin from order time of each NEC case (infant is blue circle, number=minutes), displayed by outcome group (surgery or no surgery). Outliers from the surgical group 367 and 389 did not receive any other antibiotics before this time. Outlier 304 in non-surgical group received other antibiotics (nafcillin and gentamicin) and had HSV+ cultures. (Plot of Vancomycin Delay in Minutes by Medical or Surgical NEC Group).

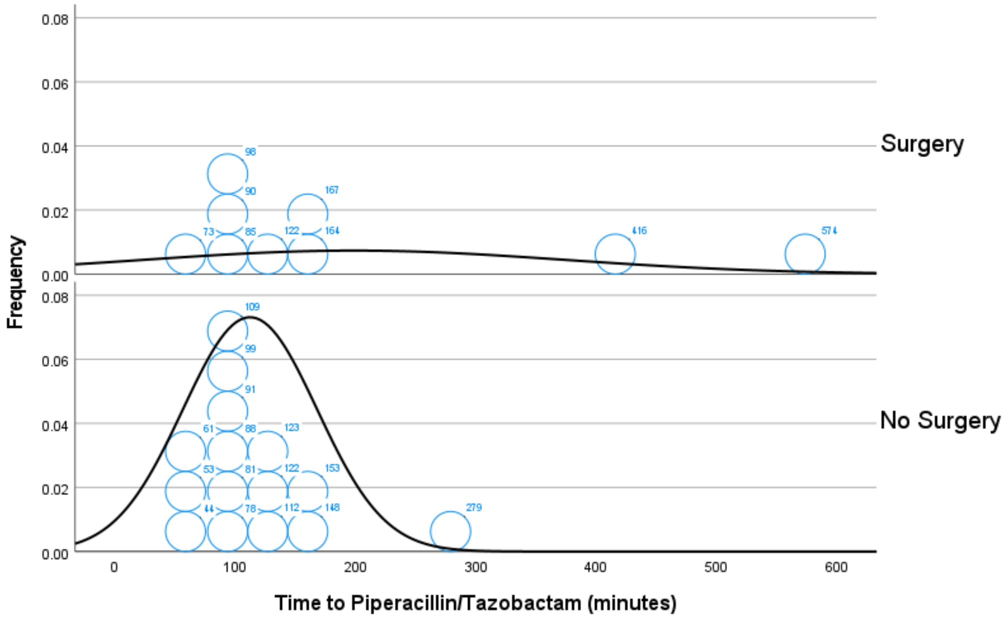

Figure S2. Histogram of minutes to piperacillin/tazobactam from order time of each NEC case (infant is blue circle, number=minutes), displayed by outcome group (surgery or no surgery). Outliers in the surgical group 416, and 574 did not receive antibiotics before given pip/taz (excluding vancomycin). Outlier 279 in the non-surgical group received nafcillin and gentamicin. (Plot of Piperacillin/tazobactam Delay in Minutes by Medical or Surgical NEC Group).

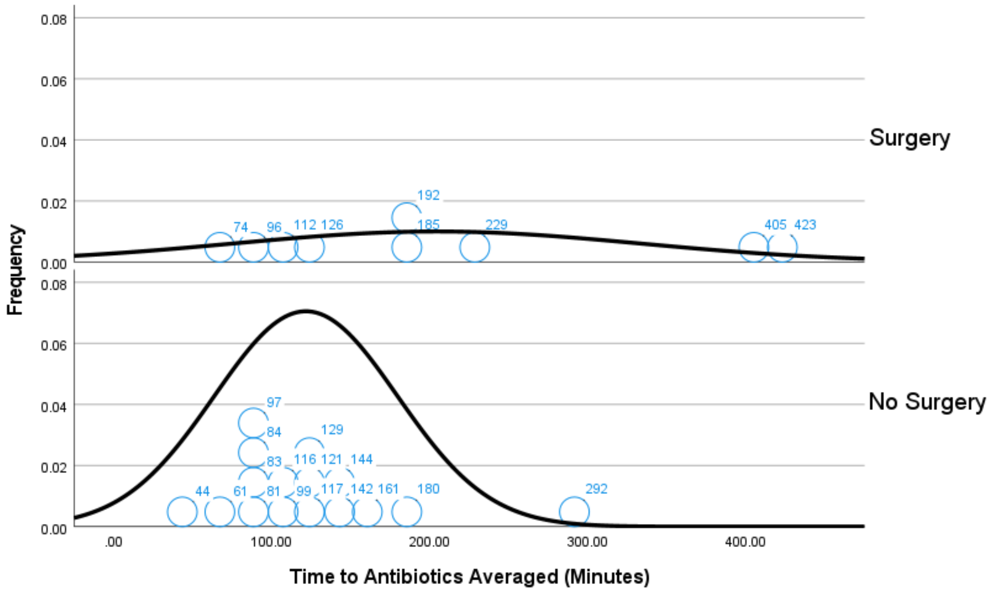

Figure S3. Histogram of average minutes to antibiotics (vancomycin and piperacillin/tazobactam) from order time of each NEC case (infant is blue circle, number=minutes), displayed by outcome group (surgery or no surgery). (Plot of Average Antibiotic Delay in Minutes per Infant by Medical or Surgical NEC Group).
